# Persistent Umbilical Discharge from an Omphalomesenteric Duct Cyst Containing Gastric Mucosa

**DOI:** 10.1155/2012/482185

**Published:** 2012-05-24

**Authors:** Kanimozhi Tamilselvan, Arunodaya Mohan, Sarah Cheslyn-Curtis, Michael Eisenhut

**Affiliations:** Department of Paediatrics, Luton and Dunstable Hospital NHS Foundation Trust, Lewsey Road, Luton LU4 0DZ, UK

## Abstract

Umbilical discharge in infancy is often attributed to infection or an umbilical granuloma. It is important to investigate if such a discharge is due to an underlying congenital abnormality because corrective surgical intervention may then be required. We present the first case of an infant with a persistent umbilical discharge from an omphalomesenteric duct cyst. The discharge was associated with periumbilical dermatitis. The dermatitis was most likely due to irritation of the skin by gastric acid produced by the ectopic gastric mucosa contained in the omphalomesenteric duct cyst. Both discharge and dermatitis resolved after surgical removal of the cyst.

## 1. Introduction

Umbilical disorders can result from failure of an embryologic process. Basic understanding of the anatomy and embryology is necessary to identify and treat umbilical disorders. The omphalomesenteric duct (OMD) appears at the beginning of embryonic life as a long, tubular structure that connects the midgut to the yolk sac. The OMD normally regresses during the 5th−9th weeks of fetal development, leaving a solid cord that runs from the ileum to the umbilicus. If the lumen of the OMD has not completely disappeared at birth, various abnormalities can result including a fistula between the ileum and the umbilicus, an omphalomesenteric duct sinus emerging from the umbilicus and blind ending, a cyst representing a remnant of a patent segment of the OMD, a diverticulum whose enteric portion is patent, that is, Meckel's diverticulum, or an umbilical polyp [1].

Approximately two percent of the population may have an OMD remnant, and the commonest variant is an asymptomatic Meckel's diverticulum. We present the case of an umbilical cyst lined with gastric mucosa discharging from the umbilicus through a sinus with fistula.

## 2. Case Report

A 6-month-old female infant had presented five times to medical services since birth with a history of discharge from the umbilicus. On days 2 and 4, she presented with bleeding from the umbilical stump, which was managed with a pressure dressing. At 2 weeks of age, she attended a general practitioner surgery with discharge of watery liquid from the umbilicus, and it was treated like an umbilical granuloma with silver nitrate application. She came to the paediatric assessment unit of the local district general hospital at 3 weeks of age because of ongoing discharge. The periumbilical skin area appeared raw with features of a chemical dermatitis. An umbilical swab was taken and treatment with oral flucloxacillin commenced. The child presented again with ongoing symptoms. This time, it was diagnosed as excoriated umbilical granuloma with possible tinea corporis infection and she was treated with miconazole and neomycin. The discharge was mainly serous fluid which occasionally turned serosanguinous but did not contain pus.

**Figure 1:**
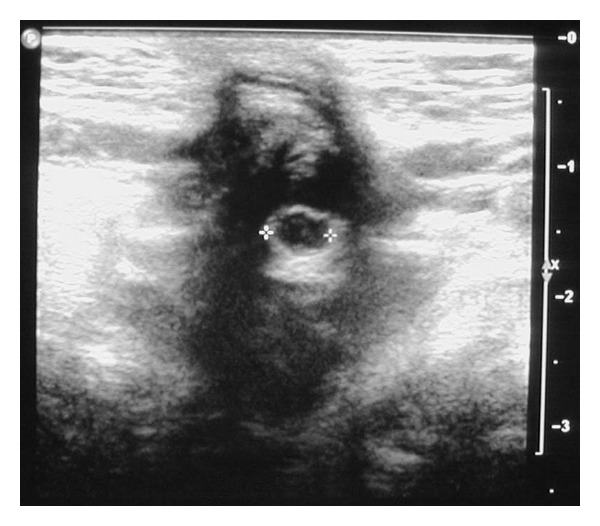
Umbilical cyst visualized by ultrasound through the anterior abdominal wall.

 At 6 months of age, she was rereferred by the general practitioner for persistent umbilical discharge with intermittent blood-stained fluid whilst she had been on her third course of antibiotics. On this occasion a consultant paediatrician reviewed the infant. An ultrasound was requested to investigate for remnants of an omphalomesenteric duct because of the persistence of symptoms. The ultrasound showed a cystic structure of 5 mm diameter with echogenic walls situated deep to the umbilicus (see Figure 1).

**Figure 2:**
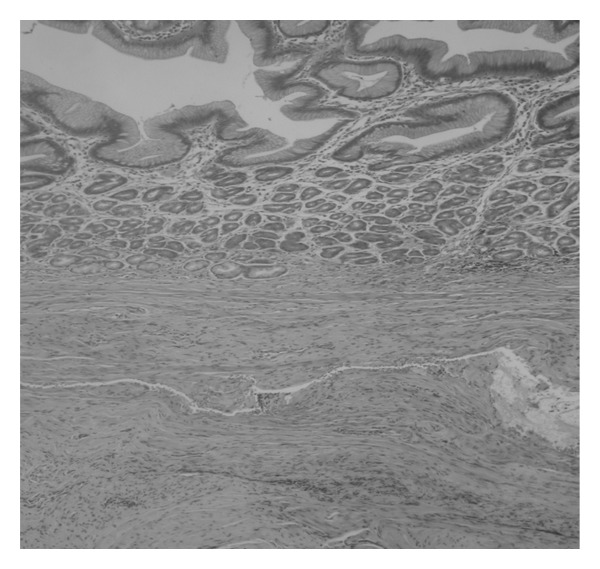
Umbilical cyst lined by ectopic gastric mucosa.

There was no communication with the bowel. A fistula extended through the anterior abdominal wall to the umbilicus. She was referred to surgeons for further management. She had a surgical excision of the cyst and sinus tract at 7 months. On macroscopic analysis, it was found to be a hard cystic lesion below the umbilicus surrounded by thick chronic inflammatory tissue. A fistula extended through the abdominal wall with no obvious communication or tract to the peritoneal cavity except for its continuation into the ligamentum teres. Histology showed skin with exudates and ulceration overlying a cystic structure lined by gastric corpus type mucosa in keeping with an umbilical cyst lined by ectopic gastric mucosa (Figure 2). Her postoperative recovery was uneventful, discharge and dermatitis resolved and she was discharged from hospital care.

## 3. Discussion

OMD remnants can present as umbilical anomalies, intestinal obstruction, acute abdomen, and painless rectal bleeding. Umbilical anomalies normally present in infancy and other features in later childhood. A study of 217 children with OMD anomalies demonstrated that approximately 40% of these lesions were symptomatic, and among these, 80% presented in first 2 years of life [2]. In another retrospective study, 59 children presenting with a symptomatic OMD remnant during a 17-year period at a tertiary pediatric surgery unit were reviewed [3]. Patients presented with gastrointestinal tract obstruction in 36%, with acute abdomen in 31%, with umbilical abnormalities in 29%, and rectal bleeding in 5%. In 31%, ectopic tissue was detected which in 25% was gastric mucosa. Patients with umbilical abnormalities had in 5/17 prolapse, 5/17 faecal drainage, 4/17 an umbilical polypoid mass, and in 3/17 umbilical cord hernias that contained a Meckel's diverticulum. Umbilical cysts as OMD remnants have rarely been reported. The literature review revealed 2 cases of umbilical cysts, one in a 6-year-old child presenting as an umbilical mass [4] and another in a 2-year-old girl presenting as an umbilical nodule [5]. Our paper is to our knowledge the first of a child presenting with persistent discharge as manifestation of an umbilical cyst.

 The presence of ectopic mucosa within an OMD remnant has been described before. The frequency of detecting ectopic tissue is considered to be higher in symptomatic cases. The most commonly encountered ectopic tissues are of gastric or pancreatic origins, although other tissues like colonic mucosa can also be seen [3]. This is a property shared with gastrointestinal tract duplications. The findings in our case can be distinguished from a spherical duplication by the fact that unlike in duplications our case was not in proximity to a part of the gastrointestinal tract and did not have smooth muscle in its wall [6]. The acidity of this discharge may have caused the skin appearance of a chemical dermatitis observed in our case. Ectopic gastric mucosa has been shown to produce physiologically effective amounts of acid leading to inflammation and ulceration, which may explain the blood staining of the discharge observed in our case. Previous papers documented symptoms of abdominal pain due to ectopic gastric mucosa in the gall bladder, rectum, or appendix mimicking appendicitis [7]. Umbilical discharge alone can be a symptom of varied pathology [8] (see Table 1). The commonest cause of umbilical discharge is umbilical granuloma, and it is treated with silver nitrate application. If there are persistent symptoms despite this intervention, other differential diagnoses like patent urachus and omphalomesenteric duct remnants should be thought of early and relevant investigations ordered. This should include ultrasound and Meckel's scan. Ultrasound is the first-line investigation to detect congenital anomalies affecting the umbilicus [9], and it can guide appropriate management as shown by our paper.

## Figures and Tables

**Table 1:** Differential diagnosis of umbilical discharge.

Cause	Age at presentation	Diagnostic features
Omphalitis	Neonatal	Purulent discharge Periumbilical skin inflammation
Umbilical granuloma	Neonatal	Homogenous granuloma with discharge
Umbilical hernia ulceration	Neonatal	Skin breakdown and purulent discharge with or without feculent material
Patent urachus, urachal cyst	Any age	Urine in discharge (in patent urachus), presence of second lumen in umbilical cord, purulent discharge, and mass
Patent omphalomesenteric duct remnants	Neonatal to early childhood	Serosanguinous, feculent, or bilous discharge, presence of second lumen in umbilical cord
